# Supplementary material for: Pan-Genome-Wide Association Study of Serotype 19A Pneumococci Identifies Disease-Associated Genes
Source: Microbiol Spectr. 2023 Jun 26;11(4):e04073-22. doi: 10.1128/spectrum.04073-22 (PMC10433855; doi:10.1128/spectrum.04073-22)
Supplement: Supplemental File 1 — Legends to Tables S1 to S6. Download spectrum.04073-22-s0001.pdf, PDF file, 0.1 MB [file spectrum.04073-22-s0001.pdf]

Supplementary information to:

Pan-genome-wide association study of serotype 19A pneumococci identifies disease-associated genes

Ting Li, Jiayin Huang, Shimin Yang, Jianyu Chen, Zhenjiang Yao, Minghao Zhong, Xinguang Zhong, Xiaohua Ye

Supplementary tables:

Supplementary Table S1. Basic information for *S. pneumoniae* serotype 19A isolates in this study.

Supplementary Table S2. The disease-associated genes identified by Scoary.

Supplementary Table S3. The disease-associated genes identified by LMM.

Supplementary Table S4. The disease-associated genes identified by Random Forest.

Supplementary Table S5. The sequence of the disease-associated genes.

Supplementary Table S6. The annotation of the disease-associated genes.
